# Lateral Tibial Slope on Pre-Reconstruction MRI in ACL Graft Rerupture: Association, Discriminative Performance, and Measurement Reliability

**DOI:** 10.3390/diagnostics16213476

**Published:** 2026-10-24

**Authors:** Oğuzhan Pekince, Ahmet Baytok, Alper Kırılmaz, Ahmet Faruk Çamcı, Ahmet Yıldırım

**Affiliations:** 1 Department of Orthopedics and Traumatology, Konya City Hospital, Konya 42020, Türkiye; 2 Department of Radiology, Faculty of Medicine, Selcuk University, Konya 42130, Türkiye

**Keywords:** anterior cruciate ligament reconstruction, graft rerupture, lateral tibial slope, magnetic resonance imaging, reproducibility of results

## Abstract

**Background/Objectives:** This study evaluated the association between lateral tibial slope (LTS), measured using the Hudek method on magnetic resonance imaging (MRI) before primary anterior cruciate ligament (ACL) reconstruction, and ACL graft rerupture, and assessed the discriminative performance and reliability. **Methods:** This retrospective comparative study included 23 patients with definite ACL graft rerupture and 46 controls without rerupture after at least two years of follow-up. LTS was independently measured twice, four weeks apart, by a radiologist and an orthopedic surgeon blinded to group allocation and clinical outcomes. Reliability was assessed using intraclass correlation coefficients (ICCs), interobserver agreement using Bland–Altman analysis, and discriminative performance using receiver operating characteristic (ROC) analysis. Age-adjusted associations with rerupture were evaluated using logistic regression. **Results:** LTS was significantly higher in the rerupture group in both measurement rounds for both observers (all *p* ≤ 0.009). Intraobserver repeatability was excellent for the radiologist (ICC = 0.985) and good for the orthopedic surgeon (ICC = 0.852), while interobserver agreement was moderate (ICC = 0.547 for mean measurements). The orthopedic surgeon measured LTS 0.85° higher on average. The area under the ROC curve was 0.794 (95% CI, 0.678–0.910) for the radiologist and 0.700 (95% CI, 0.563–0.837) for the orthopedic surgeon. Exploratory thresholds of ≥4.3° and ≥5.4° showed sensitivities of 60.9% and 43.5% and specificities of 93.5% and 91.3%, respectively. After age adjustment, each 1° increase in LTS was associated with 1.682-fold and 1.412-fold higher odds of rerupture (both *p* ≤ 0.003). **Conclusions:** Higher LTS before primary ACL reconstruction was associated with graft rerupture, including after age adjustment. Observer-related differences may influence absolute measurements and derived thresholds. Given their high specificity but limited sensitivity, these exploratory thresholds should not be considered validated clinical decision cutoffs.

## 1. Introduction

Anterior cruciate ligament (ACL) injuries are common orthopedic injuries that compromise knee stability and function, particularly in young and active individuals. Anterior cruciate ligament reconstruction (ACLR) is widely performed to restore knee stability and function in appropriately selected patients with symptomatic instability [1,2]. Despite advances in surgical techniques, anatomic tunnel placement, and graft fixation, ACL graft failure and rerupture remain clinically relevant concerns [1,2,3]. Because revision ACLR following graft failure may be associated with poorer clinical outcomes and a greater burden of intra-articular pathology than primary reconstruction, identifying modifiable and non-modifiable factors associated with graft failure remains important [3,4,5].

The etiology of ACL graft failure is multifactorial and involves the interplay of reinjury, surgical and technical factors, biological graft healing, and patient-specific anatomical characteristics [2,3,6]. Among these anatomical factors, posterior tibial slope (PTS) has received increasing attention in recent years [2,7]. Biomechanical studies have shown that increased PTS can increase anterior tibial translation and loading of the ACL graft under axial loading [8,9].

Because the sagittal geometry of the medial and lateral tibial plateaus differs, compartment-specific assessment of posterior tibial slope is important. In particular, growing evidence suggests that increased lateral posterior tibial slope (LTS/LPTS) is associated with primary ACL injury and graft failure [2,4,5,10]. The two-circle method described by Hudek et al. enables determination of the proximal tibial anatomic axis on MRI and standardized measurement of tibial plateau slope [11]. However, the literature remains heterogeneous regarding the magnitude of the association between increased LTS and graft failure and the threshold values that may be clinically relevant. Variability in the thresholds reported across studies may reflect differences in study populations, imaging modalities, anatomical references, and measurement techniques [3,4,5,10,12].

The multifactorial nature of graft rerupture makes it difficult to evaluate the contribution of intrinsic anatomical factors such as LTS in isolation. Therefore, comparing LTS measured on MRI before primary ACL reconstruction between patients with graft rerupture and those without rerupture may help clarify the discriminative value of this anatomical parameter. In addition, observer-related variability in LTS measurement is relevant to its clinical interpretation. Systematic measurement differences between observers from different specialties may affect not only measurement agreement but also the discriminative thresholds derived from the same dataset.

The primary aim of this study was to evaluate differences in LTS, measured using the Hudek method on MRI before primary ACL reconstruction, between patients with definite ACL graft rerupture and those without rerupture, and to assess its ability to discriminate between the two groups, including the exploratory identification of LTS thresholds. The secondary aim was to evaluate the intraobserver repeatability and interobserver agreement of repeated LTS measurements performed by a radiologist and an orthopedic surgeon. We hypothesized that LTS would be higher in patients with graft rerupture than in controls, that this association would persist after adjustment for age, and that observer-related differences in LTS measurements might be present.

## 2. Materials and Methods

### 2.1. Study Design and Ethical Approval

This retrospective, two-group comparative study was designed to evaluate the association between lateral tibial slope (LTS), measured on magnetic resonance imaging (MRI) obtained before primary anterior cruciate ligament (ACL) reconstruction, and ACL graft rerupture during follow-up. The study was approved by the Non-Interventional Clinical Research Ethics Committee of Konya City Hospital (Decision No. 2026/151; 12 May 2026) and was conducted in accordance with the principles of the Declaration of Helsinki.

### 2.2. Patient Selection and Study Groups

The electronic medical records and imaging archive of our institution were retrospectively reviewed for the period between August 2020 and December 2024.

### 2.3. Rerupture Group

Screening of hospital records and MRI reports identified 41 patients with reported ACL graft rerupture. Of these, 26 had undergone revision ACL reconstruction at our institution. Among these patients, 16 had knee MRI examinations obtained before primary ACL reconstruction available in our institutional imaging archive and were included in the study. The remaining 10 patients were excluded because their pre-reconstruction MRI examinations had been obtained at outside institutions and were therefore not available in our institutional imaging archive.

In addition, patients who had been diagnosed with ACL graft rerupture and recommended revision surgery but either declined surgery or underwent revision ACL reconstruction at another institution were evaluated. Of these patients, seven had MRI examinations obtained before primary ACL reconstruction available at our institution and were included. All seven patients had presented with recurrent symptomatic instability and had clinical examination and MRI findings consistent with graft rerupture. Revision ACL reconstruction was recommended for all seven patients; three declined the recommended procedure, as documented in their medical records, whereas four were subsequently documented through the Turkish national electronic health record system as having undergone revision ACL reconstruction at another institution. Thus, the rerupture group comprised a total of 23 patients aged 18–40 years.

Revision surgery at our institution was not required for inclusion in the rerupture group. Graft rerupture was diagnosed based on the combined assessment of patient-reported symptomatic instability, clinical evidence of instability on physical examination (anterior drawer, Lachman, and/or pivot-shift tests), and MRI findings consistent with ACL graft rerupture. Patients who underwent revision ACL reconstruction or for whom revision surgery was recommended following this assessment were included in the rerupture group. Cases in which graft rerupture was considered suspicious or could not be definitively classified based on the combined clinical and imaging findings were excluded.

### 2.4. Control Group

The control group was drawn from patients aged 18–40 years who had undergone primary ACL reconstruction during the same study period, had knee MRI examinations obtained before primary surgery available in our institutional imaging archive, had a minimum follow-up of two years, and had no graft rerupture identified during follow-up. A total of 94 patients with at least two years of documented postoperative follow-up were assessed for control eligibility. Eighteen patients were excluded because their pre-reconstruction MRI examinations had been obtained outside our institution. Of the remaining 76 patients, 22 were excluded because they were older than 40 years, leaving 54 eligible control candidates. To achieve the prespecified 1:2 case-to-control ratio, 46 of these 54 eligible patients were randomly selected by lot drawing for inclusion in the control group, while eight were not selected. Follow-up was ascertained through institutional electronic medical records and documented postoperative outpatient visits. Among patients meeting these criteria, 46 controls were randomly selected. Controls were not individually matched to rerupture cases by age or sex.

**Figure 1:**
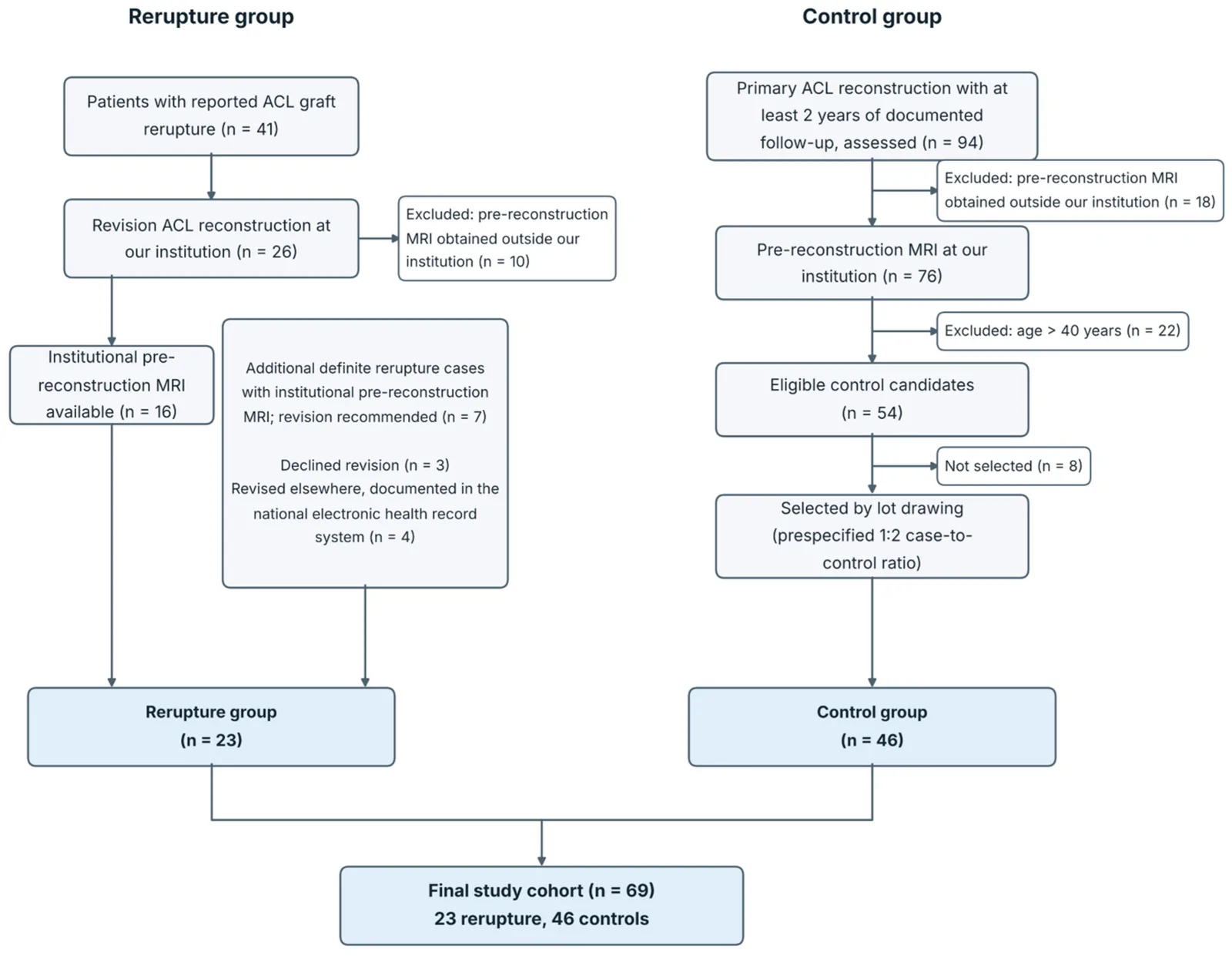
Flow diagram of patient selection and study-group formation. ACL, anterior cruciate ligament; MRI, magnetic resonance imaging.

The final study cohort comprised 69 patients, including 23 patients with definite graft rerupture and 46 controls (Figure 1).

### 2.5. Inclusion and Exclusion Criteria

Patients aged 18–40 years who had undergone primary ACL reconstruction and had knee MRI examinations obtained before primary ACL reconstruction of sufficient image quality available in our institutional imaging archive were eligible for inclusion. For inclusion in the control group, a minimum of two years of documented postoperative follow-up without identified graft rerupture was required. For inclusion in the rerupture group, a definite diagnosis of ACL graft rerupture during follow-up was additionally required.

Exclusion criteria were the unavailability of pre-reconstruction MRI examinations in our institutional imaging archive, inadequate image quality precluding reliable LTS measurement, graft rerupture that could not be definitively confirmed, previous tibial osteotomy, and multiligament knee injury.

### 2.6. MRI Assessment and LTS Measurement

All LTS measurements were performed on MRI examinations obtained before primary ACL reconstruction. This approach was intended to avoid potential effects of postoperative tibial tunnels, implants, and changes related to revision surgery on the measurements. All MRI examinations were acquired at the same institution using the same 1.5-T MRI scanner (Magnetom Area; Siemens AG Medical Solutions, Forchheim, Germany) and a standardized knee MRI protocol. LTS measurements were performed on sagittal T1-weighted spin-echo images acquired with a slice thickness of 3 mm and an acquisition matrix of 320 × 320. Coronal proton-density-weighted turbo spin-echo fat-suppressed images, acquired with a slice thickness of 3 mm and an acquisition matrix of 240 × 256, were used as reference images for localization of the lateral tibial plateau.

LTS was measured using the two-circle method described by Hudek et al. [11]. First, the central sagittal section of the proximal tibia was identified as the section providing clear visualization of the proximal tibial cortical boundaries and allowing placement of the two circles according to the Hudek method. On this section, two circles were positioned within the proximal tibia to conform to the cortical boundaries. The proximal circle was positioned tangent to the anterior, posterior, and superior cortical margins. The center of the distal circle was positioned at the level of the inferior border of the proximal circle, with the circle adjusted to conform to the anterior and posterior tibial cortices. The line connecting the centers of the two circles was defined as the tibial anatomic axis (TAA).

The lateral tibial plateau was localized using the coronal proton-density–weighted turbo spin-echo fat-suppressed reference images, and the sagittal section passing through the mediolateral center of the lateral tibial plateau was selected for measurement. A reference line perpendicular to the TAA was constructed. A tangent line was then drawn along the subchondral bone surface of the lateral tibial plateau, excluding the articular cartilage. The acute angle between the plateau tangent and the reference line perpendicular to the TAA was recorded as the LTS. A posteriorly downward slope was defined as a positive value.

All image assessments and angular measurements were performed using Weasis version 3.0.3.

**Figure 2:**
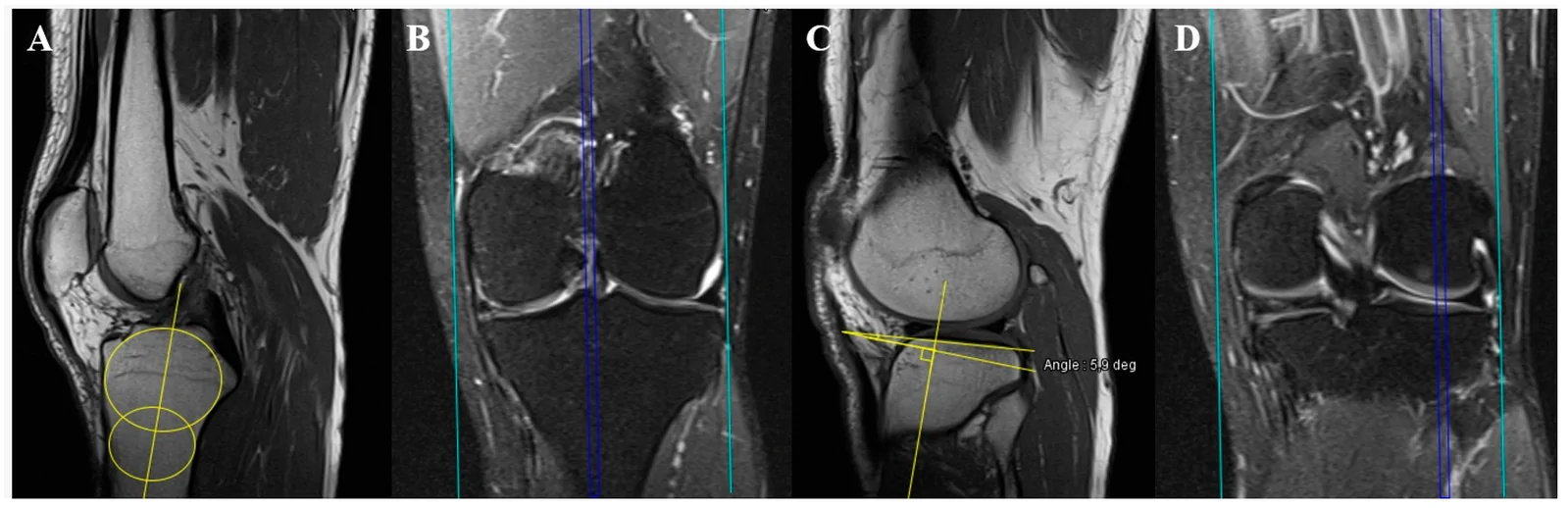
Negative lateral posterior tibial slope measured using the Hudek method. Left knee MRI of a 39-year-old man. (**A**) On a central sagittal T1-weighted image, two circles are placed within the proximal tibia. The proximal circle is fitted to the anterior, posterior, and superior cortical margins, while the distal circle is fitted to the anterior and posterior cortices, with its center positioned on the circumference of the proximal circle. The line connecting their centers defines the proximal tibial anatomical axis. The yellow circles and line indicate the Hudek two-circle construction and the resulting tibial anatomical axis. (**B**) Coronal fat-suppressed proton density-weighted image demonstrates the location of the central sagittal reference plane; the blue and cyan lines are reference/localizer lines displayed by the imaging software. (**C**) On a sagittal T1-weighted image through the center of the lateral tibial plateau, the slope is measured as the angle between a line tangent to the subchondral bone surface and a line perpendicular to the tibial anatomical axis, yielding a negative posterior tibial slope of −5.9°. The yellow lines indicate the measurement lines used to determine the slope. (**D**) Coronal fat-suppressed proton density-weighted image confirms the location of the lateral tibial plateau measurement plane; the blue and cyan lines are reference/localizer lines displayed by the imaging software.

**Figure 3:**
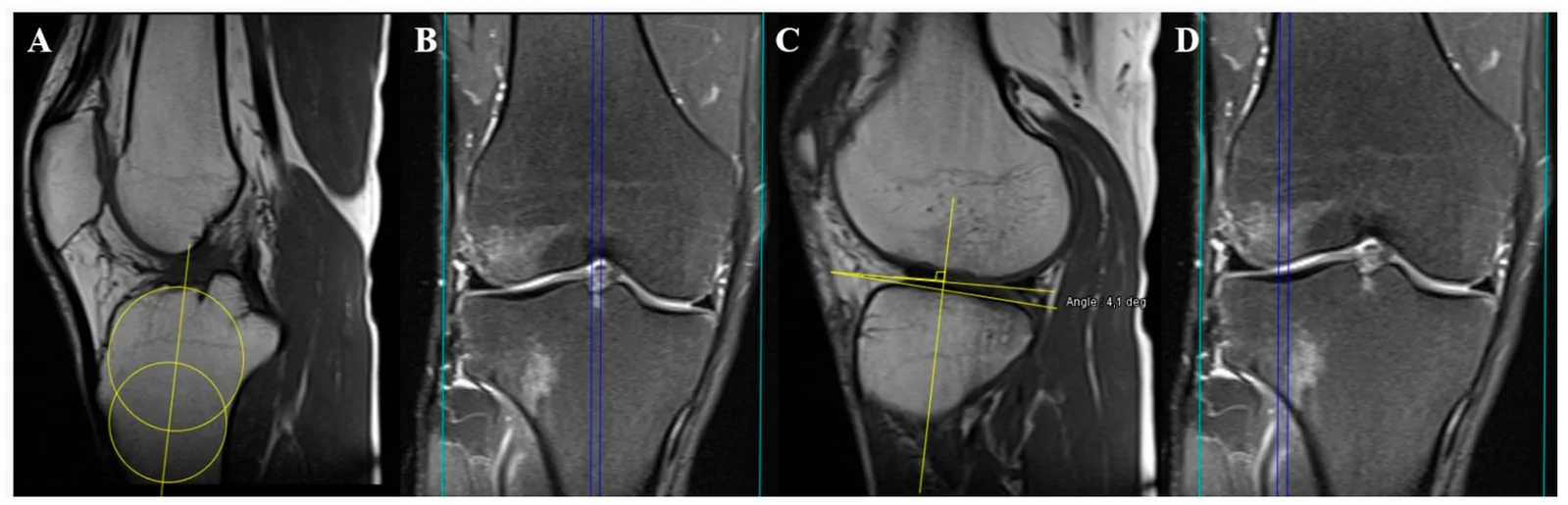
Positive lateral posterior tibial slope measured using the Hudek method, with associated bone marrow edema. Right knee MRI of a 27-year-old man. (**A**) Central sagittal T1-weighted image demonstrates the two-circle technique used to establish the proximal tibial anatomical axis. The axis is defined by connecting the centers of the proximal and distal circles fitted within the proximal tibia. The yellow circles and line indicate the Hudek two-circle construction and the resulting tibial anatomical axis. (**B**) Coronal fat-suppressed proton density-weighted image shows the location of the central sagittal reference plane; the blue and cyan lines are reference/localizer lines displayed by the imaging software. (**C**) Sagittal T1-weighted image through the center of the lateral tibial plateau demonstrates the angle between the subchondral plateau tangent and the perpendicular to the anatomical axis, yielding a positive posterior tibial slope of +4.1°. The yellow lines indicate the measurement lines used to determine the slope. (**D**) Coronal fat-suppressed proton density-weighted image confirms the location of the lateral tibial plateau measurement plane and demonstrates bone marrow edema in the lateral femoral condyle and lateral metaphyseal region of the tibia; the blue and cyan lines are reference/localizer lines displayed by the imaging software.

Representative examples illustrating the measurement technique for negative and positive LTS values are shown in Figure 2 and Figure 3, respectively.

### 2.7. Observers, Blinding, and Repeated Measurements

All LTS measurements were performed independently by a radiologist with 9 years of total professional experience in radiology and an orthopedic surgeon with 7 years of total professional experience in orthopedics and traumatology. Before measurement, the MRI examinations were exported from the hospital imaging system, anonymized, and stored on an external storage device designated for the study. All patient identifiers were removed, and images from the rerupture and control groups were arranged in mixed order. The images and the data file containing the measurement records were linked exclusively using anonymized study-specific identification numbers. During the measurement process, the observers were prevented from accessing the patients’ medical records and were therefore blinded to clinical characteristics, follow-up outcomes, and rerupture or control group status.

Each observer performed LTS measurements for all cases in two separate assessment rounds. After completion of the first assessment, the measurement results were submitted to the investigator, and the observers’ own records of the initial measurements were deleted. The second assessment was performed 4 weeks after the first, without access to the previous measurement results. The same anonymized image set was used in both assessment rounds.

Measurements obtained by each observer across the two assessment rounds were used to evaluate intraobserver repeatability. Measurements performed by the radiologist and orthopedic surgeon in the same cases were used to assess interobserver agreement. The rerupture and control groups were compared separately for the first and second measurements of each observer. For the ROC and logistic regression analyses, the mean of the two repeated LTS measurements obtained by each observer was used.

### 2.8. Variables and Outcomes

The primary independent variable was LTS measured on MRI obtained before primary ACL reconstruction. The primary outcome was the presence of definite ACL graft rerupture during follow-up.

In the primary analysis, LTS was treated as a continuous variable, and differences in LTS between the rerupture and control groups were evaluated. The ability of LTS to discriminate between the two groups was also assessed using receiver operating characteristic (ROC) analysis, and potential discriminative thresholds were identified on an exploratory basis.

Secondary outcomes were the intraobserver repeatability of repeated measurements performed by the radiologist and orthopedic surgeon and the interobserver agreement between the two observers. Age, sex, side, and concomitant surgical procedures performed during primary ACL reconstruction were obtained from the patient records. Other potentially relevant factors, including reinjury mechanism, activity level, and objective measures of tunnel position or malposition, were not systematically evaluated across the entire cohort.

### 2.9. Sample Size and Statistical Power

The statistical power of the available sample was evaluated using G*Power version 3.1.9.7. Based on the values reported by Jaecker et al. [6], a Cohen’s *d* effect size of 0.863 was calculated. Using a two-sided independent-samples *t*-test with an α level of 0.05 and the current sample of 23 patients with rerupture and 46 controls, the statistical power of the available sample to detect a group difference in the magnitude reported in the reference study was calculated to be 91.5%.

This retrospective power calculation was intended only to characterize the statistical power of the available sample for the primary between-group comparison of LTS and should not be interpreted as establishing adequate precision for the ROC-derived thresholds, logistic regression estimates, or reliability analyses. The precision of these secondary estimates was therefore evaluated primarily through their corresponding confidence intervals.

### 2.10. Statistical Analysis

Statistical analyses were performed using SPSS (Statistical Package for the Social Sciences), version 18.0. Categorical variables were summarized as numbers and percentages, whereas continuous variables were summarized using means ± standard deviations and medians (first–third quartiles). The normality of continuous variables was assessed using the Kolmogorov–Smirnov test. For comparisons between two independent groups, the independent-samples *t*-test was used for normally distributed continuous variables, and the Mann–Whitney U test was used for non-normally distributed variables. Categorical variables were compared using the chi-square test or Fisher’s exact test, as appropriate.

The unit of analysis for reliability assessment was the patient-level LTS measurement, expressed in degrees. Intraobserver repeatability was assessed by comparing the two measurement rounds of each observer across patients, whereas interobserver agreement was assessed by comparing patient-level LTS measurements between the two observers. Intraobserver and interobserver measurement reliability for the radiologist and orthopedic surgeon was assessed using the intraclass correlation coefficient (ICC) based on a two-way mixed-effects model, absolute agreement, and single measurements. ICC values were reported with 95% confidence intervals (CIs). ICC values were interpreted as poor (<0.50), moderate (0.50–0.75), good (0.75–0.90), and excellent (>0.90) [13].

Agreement between the mean values of the two repeated measurements obtained by the radiologist and orthopedic surgeon was further assessed using Bland–Altman analysis. Measurement differences were calculated as the radiologist’s mean measurement minus the orthopedic surgeon’s mean measurement. The normality of the differences was assessed using the Kolmogorov–Smirnov test. The 95% limits of agreement were calculated as the mean difference ± 1.96 × the standard deviation of the differences. Potential proportional bias was assessed by linear regression of the differences between observers on the mean of the two observers’ measurements.

The ability of the mean of the two repeated LTS measurements obtained by each observer to discriminate between the rerupture and control groups was evaluated using receiver operating characteristic (ROC) curve analysis. The area under the curve (AUC) was reported with its 95% CI. A potential discriminative threshold for each observer was determined using the Youden index (sensitivity + specificity − 1). Sensitivity, specificity, positive predictive value (PPV), and negative predictive value (NPV) were calculated at the identified thresholds. The 95% CIs for sensitivity and specificity were calculated using the exact binomial (Clopper–Pearson) method. Youden-derived thresholds obtained from the same dataset were considered exploratory and were not interpreted as validated clinical decision thresholds.

The association between LTS and graft rerupture was evaluated using multivariable logistic regression analysis. Separate models were constructed for the radiologist and orthopedic surgeon. In each model, the mean of the observer’s two LTS measurements and age were entered simultaneously, with both variables treated as continuous variables. Results were reported as age-adjusted odds ratios (ORs) for each 1° increase in LTS, with 95% CIs. Given the limited number of rerupture events, the models were intentionally restricted to LTS and age to reduce the risk of overfitting. Accordingly, the resulting estimates were interpreted as age-adjusted associations rather than evidence of an independent prognostic effect.

All statistical tests were two-sided, and *p* < 0.05 was considered statistically significant.

### 2.11. Use of Generative Artificial Intelligence

Generative artificial intelligence was used during manuscript preparation and revision to assist with language refinement, improvement of textual clarity and organization, and editorial revision. The AI tool was not used for patient selection, clinical outcome classification, MRI measurements, generation or modification of study data, or statistical analysis. All AI-assisted text was critically reviewed, verified, and revised by the authors, who take full responsibility for the final content of the manuscript.

## 3. Results

A total of 69 patients who had undergone primary anterior cruciate ligament (ACL) reconstruction were included in the study, comprising 23 patients who experienced graft rerupture during follow-up and 46 controls without rerupture. The rerupture group was numerically younger than the control group (median age, 28 vs. 37 years), although this difference did not reach statistical significance (*p* = 0.103). No statistically significant differences were observed between the groups in sex, operated side, or concomitant surgical procedures (all *p* > 0.05) (Table 1).

Table 2 compares the LTS measurements obtained by the radiologist and orthopedic surgeon between the rerupture and control groups. The radiologist’s first and second measurements were significantly higher in the rerupture group than in the control group (both *p* < 0.001). Similarly, the orthopedic surgeon’s first and second measurements were significantly higher in the rerupture group than in the control group (*p* = 0.009 and *p* = 0.002, respectively).

When intraobserver measurement reliability was evaluated, excellent repeatability was observed between the radiologist’s first and second measurements (ICC = 0.985; 95% CI, 0.975–0.990), whereas good repeatability was observed between the orthopedic surgeon’s first and second measurements (ICC = 0.852; 95% CI, 0.769–0.906) (Table 3).

Moderate interobserver agreement was observed between the radiologist and orthopedic surgeon for both the first measurements (ICC = 0.514; 95% CI, 0.311–0.671) and the second measurements (ICC = 0.541; 95% CI, 0.352–0.688). Agreement between the two observers was also moderate when the mean of the two repeated measurements for each observer was compared (ICC = 0.547; 95% CI, 0.355–0.694) (Table 4).

**Figure 4:**
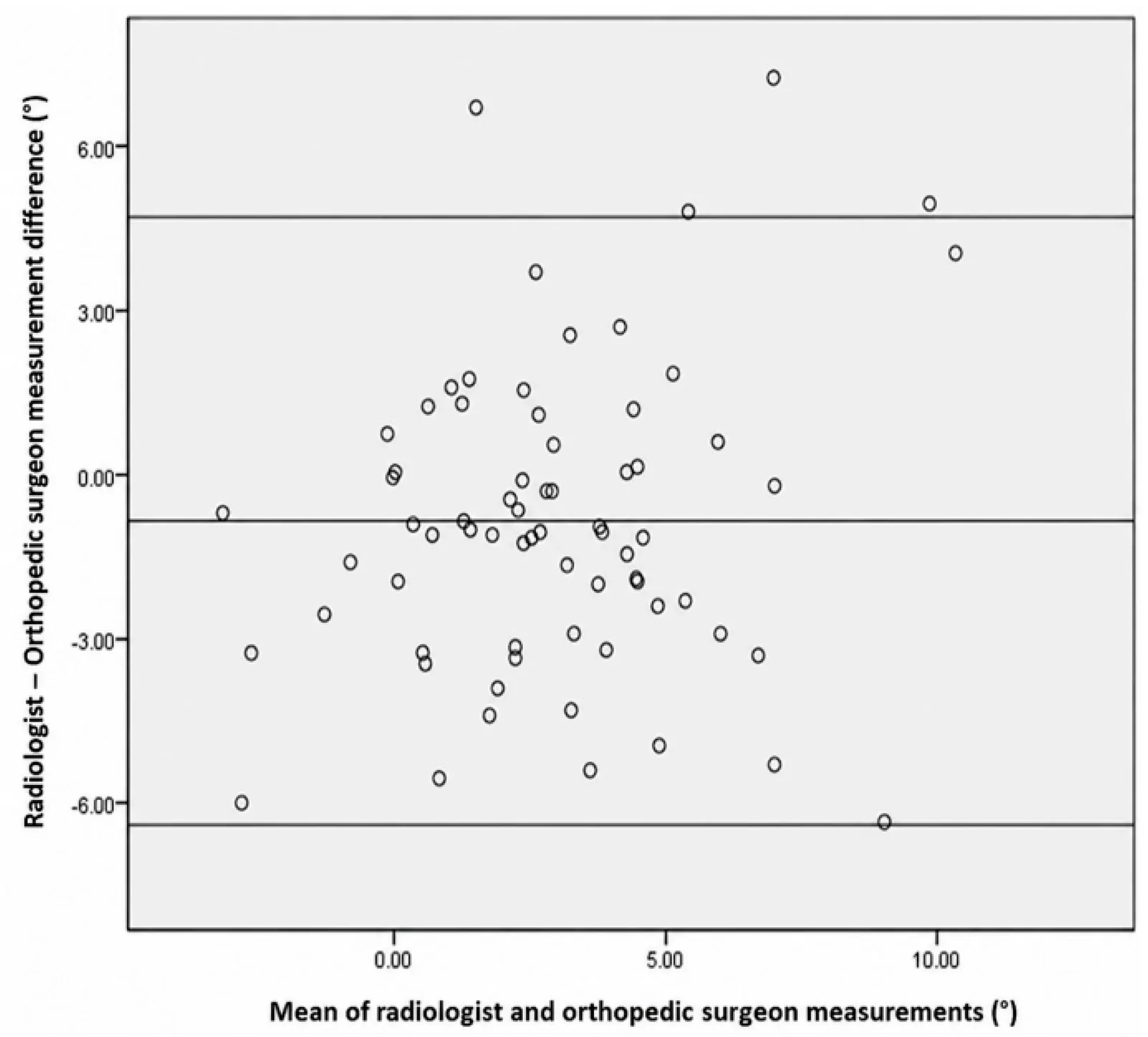
Bland–Altman plot of the mean measurements obtained by the radiologist and orthopedic surgeon.

Agreement between the mean values of the two repeated measurements obtained by the radiologist and orthopedic surgeon was evaluated using Bland–Altman analysis. The mean difference between observers was −0.85 ± 2.83°, with 95% limits of agreement ranging from −6.40° to 4.70°. Accordingly, the orthopedic surgeon’s measurements were, on average, 0.85° higher than those of the radiologist. No significant deviation from normality was observed for the measurement differences. Linear regression analysis evaluating the relationship between the measurement differences and the mean of the observers’ measurements showed no significant proportional bias (B = 0.202; *p* = 0.116). Because a clinically acceptable measurement difference was not predefined, these findings were insufficient to establish that the measurements could be used interchangeably (Figure 4).

**Figure 5:**
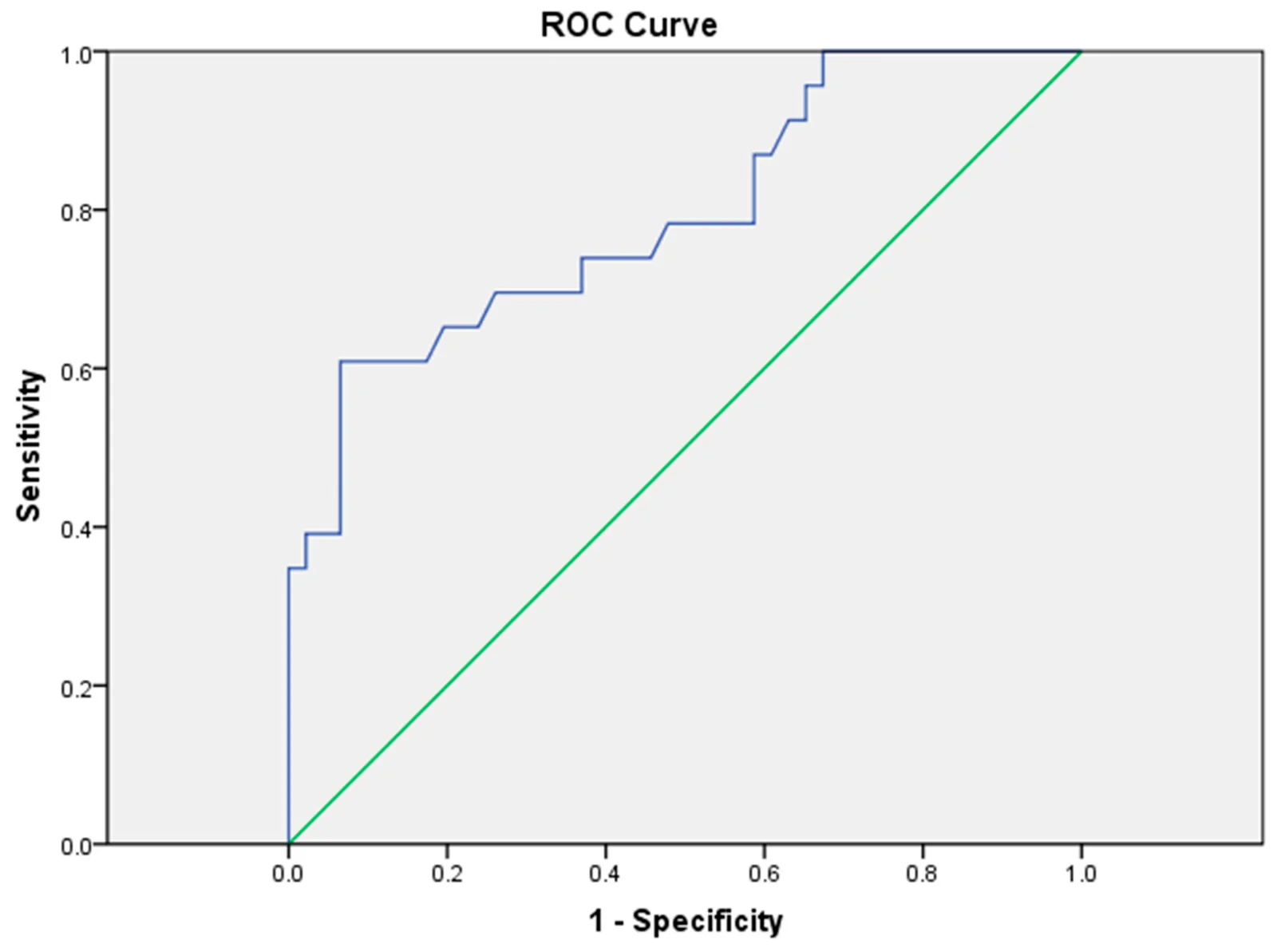
ROC curve for the ability of the radiologist’s mean of two measurements to discriminate rerupture. The blue line represents the ROC curve, and the green diagonal line represents the reference line corresponding to no discriminative ability.

**Figure 6:**
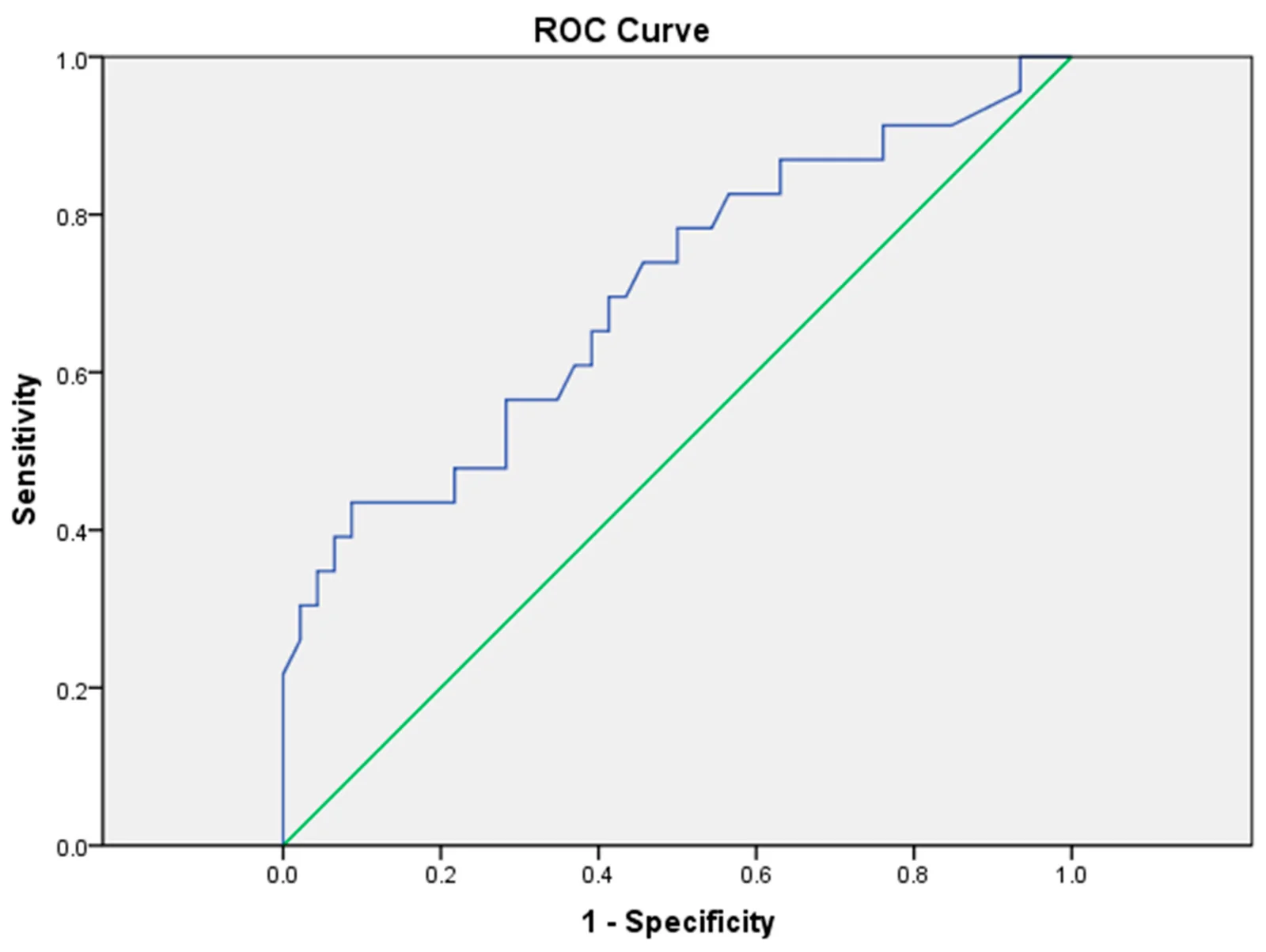
ROC curve for the ability of the orthopedic surgeon’s mean of two measurements to discriminate rerupture. The blue line represents the ROC curve, and the green diagonal line represents the reference line corresponding to no discriminative ability.

The ability of the mean of the two repeated measurements obtained by the radiologist and orthopedic surgeon to discriminate between the rerupture and control groups was evaluated using ROC analysis (Figure 5 and Figure 6). The AUC was 0.794 (95% CI, 0.678–0.910; *p* < 0.001) for the radiologist and 0.700 (95% CI, 0.563–0.837; *p* = 0.007) for the orthopedic surgeon. According to the Youden index, at a cutoff value of ≥4.3° for the radiologist, sensitivity was 60.9%, specificity was 93.5%, positive predictive value (PPV) was 82.4%, and negative predictive value (NPV) was 82.7%. For the orthopedic surgeon, at a cutoff value of ≥5.4°, the corresponding values were 43.5%, 91.3%, 71.4%, and 76.4%, respectively. The Youden index was 0.543 for the radiologist and 0.348 for the orthopedic surgeon (Table 5).

The mean of the two repeated measurements obtained by the radiologist and orthopedic surgeon was evaluated in separate logistic regression models that included age. In the model including age and the radiologist’s mean LTS measurement, each 1° increase in LTS was associated with 1.68-fold higher odds of rerupture (adjusted OR = 1.682; 95% CI, 1.241–2.280; *p* = 0.001). Similarly, in the model including age and the orthopedic surgeon’s mean LTS measurement, each 1° increase in LTS was associated with 1.41-fold higher odds of rerupture (adjusted OR = 1.412; 95% CI, 1.123–1.776; *p* = 0.003). Age was not significantly associated with rerupture in the model based on the radiologist’s measurements (OR = 0.930; 95% CI, 0.860–1.006; *p* = 0.071). In the model based on the orthopedic surgeon’s measurements, increasing age was associated with lower odds of rerupture (OR = 0.923; 95% CI, 0.856–0.995; *p* = 0.036) (Table 6).

## 4. Discussion

In this study, lateral tibial slope (LTS), measured using the Hudek method on MRI obtained before primary ACL reconstruction, was significantly higher in patients who developed graft rerupture than in those without rerupture. This difference was observed in both measurement rounds performed by both the radiologist and the orthopedic surgeon, and the association between LTS and rerupture persisted after adjustment for age. Each 1° increase in LTS was associated with 1.68-fold higher odds of rerupture based on the radiologist’s measurements and 1.41-fold higher odds based on the orthopedic surgeon’s measurements. The discriminative performance of LTS yielded an AUC of 0.794 for the radiologist and 0.700 for the orthopedic surgeon, with the exploratory thresholds demonstrating high specificity but low-to-moderate sensitivity for both observers. In addition, intraobserver repeatability was good to excellent (ICC, 0.852–0.985), whereas interobserver agreement remained moderate (ICC, 0.514–0.547). Taken together, these findings suggest that LTS may represent an anatomical feature associated with graft rerupture; however, observer-related variability should be considered when interpreting absolute measurements and the thresholds derived from them.

Our findings are consistent with biomechanical studies showing that increased posterior tibial slope can increase anterior tibial translation and loading of the ACL graft under axial loading [2,8,9]. Given the contribution of lateral compartment geometry to rotational knee kinematics, particular attention has been directed toward the association of LTS with ACL injury and graft failure [4,5,10]. In the present study, the finding of significantly higher LTS in the rerupture group across both measurement rounds for both observers is consistent with the direction of previous clinical and biomechanical findings. However, because medial tibial slope was not evaluated in the present study, no conclusions can be drawn regarding the relative contributions of lateral and medial tibial slope or the superiority of LTS over other tibial slope measurements. Furthermore, given the observational design of the study, the association between LTS and graft rerupture should not be interpreted as causal.

### 4.1. Absolute LTS Values and Thresholds

The absolute LTS values measured in our study were notably lower than those reported in some previous studies. In the rerupture group, mean LTS values ranged from approximately 4.6° to 5.0°, depending on the measurement round and observer, whereas values in the control group ranged from approximately 1.3° to 2.8°. Christensen et al. [4] reported a mean LTS of 8.4° in patients with early graft failure and 6.5° in controls. A similar difference is evident in the reported threshold values. Grassi et al. [5] reported a threshold of 7.4°, with 88% sensitivity and 84% specificity, whereas Ye et al. [12] reported a threshold of 7.7°, with 82.1% sensitivity and 80.4% specificity; Buyukkuscu et al. [10] identified a threshold of 6.0°. In contrast, the exploratory thresholds identified in the present study were ≥4.3° for the radiologist and ≥5.4° for the orthopedic surgeon.

The reasons for these between-study differences cannot be definitively determined from the present dataset. In addition to differences in study populations and patient selection criteria, variability in imaging protocols, definition of the tibial anatomic axis, selection of the lateral plateau section, measurement methodology, observer characteristics, and definitions of graft failure may contribute to differences in absolute LTS values. Although the two-circle method described by Hudek et al. [11] enables standardized determination of the proximal tibial anatomic axis on MRI, the present study did not directly compare different measurement methods; therefore, the lower values observed in our cohort cannot be attributed solely to the measurement method used. The between-group difference observed in our cohort (approximately 3.4–3.6° for the radiologist and 2.2° for the orthopedic surgeon) was comparable to or larger than the difference reported by Christensen et al. [4] (1.9°), despite markedly lower absolute values. This suggests that the lower absolute LTS values and thresholds in our cohort may reflect differences in measurement methodology and population rather than a weaker association, although this cannot be confirmed with the present data. Because the thresholds in the present study were derived from the same cohort in which their performance was evaluated, they should be regarded as cohort-specific exploratory values rather than alternative universal thresholds to those previously reported in the literature.

### 4.2. Discriminative Performance and Clinical Interpretation of the Thresholds

In the ROC analysis, the AUC was 0.794 (95% CI, 0.678–0.910) for the mean of the radiologist’s measurements and 0.700 (95% CI, 0.563–0.837) for the mean of the orthopedic surgeon’s measurements. These AUC values were derived from separate ROC analyses, and no direct statistical comparison between the AUCs of the two observers was performed. Therefore, no conclusion can be drawn that either observer had superior discriminative performance.

According to the Youden index, the ≥4.3° threshold for the radiologist yielded a sensitivity of 60.9% and a specificity of 93.5%, whereas the ≥5.4° threshold for the orthopedic surgeon yielded a sensitivity of 43.5% and a specificity of 91.3%. This performance profile suggests that higher LTS values may serve as a supportive anatomical feature associated with rerupture; however, given the limited sensitivity, LTS alone would not be appropriate as a screening tool or for ruling out rerupture. The high specificity should also not be interpreted as indicating that an individual patient with an LTS value above the threshold has a correspondingly high probability of rerupture. Furthermore, positive and negative predictive values depend on the proportion of patients with rerupture in the study sample, which was influenced by the group-selection process inherent in the study design. Therefore, the PPV and NPV observed in this study should not be directly generalized to clinical populations with different rerupture prevalence.

In addition, the thresholds were derived using the Youden index within the same study cohort and were not validated in an independent cohort. Because threshold derivation and performance evaluation were performed in the same relatively small cohort, the reported discriminative performance may be subject to optimism and may overestimate performance in an independent population. The relatively wide confidence intervals around the sensitivity estimates further indicate uncertainty regarding the precision of these thresholds. Therefore, the values of ≥4.3° and ≥5.4° should not be regarded as validated clinical decision thresholds or surgical indication cutoffs. The present data are insufficient to support decisions regarding additional surgical procedures, such as lateral extra-articular tenodesis or slope-modifying osteotomy, based on LTS alone. The potential role of LTS in such clinical decision-making should be evaluated alongside other patient-related, anatomical, and surgical factors and requires validation in independent cohorts.

### 4.3. Age and Multivariable Analysis

Although the median age was lower in the rerupture group than in the control group (28 vs. 37 years), the between-group difference did not reach statistical significance (*p* = 0.103). Nevertheless, age was significantly associated with rerupture in the multivariable model based on the orthopedic surgeon’s measurements (OR = 0.923; 95% CI, 0.856–0.995; *p* = 0.036), whereas it did not reach statistical significance in the model based on the radiologist’s measurements (OR = 0.930; 95% CI, 0.860–1.006; *p* = 0.071). Previous studies have reported that younger age may be associated with graft failure after ACL reconstruction [14,15,16]. In this respect, the direction of the present findings is consistent with the existing literature.

More importantly, the association between LTS and rerupture remained statistically significant for both observers after age was included in the models. Each 1° increase in LTS was associated with 1.682-fold higher odds of rerupture in the radiologist-based model (95% CI, 1.241–2.280; *p* = 0.001) and 1.412-fold higher odds in the orthopedic surgeon-based model (95% CI, 1.123–1.776; *p* = 0.003). These findings support the view that the observed association between LTS and rerupture cannot be explained solely by the age difference between the groups. However, the multivariable models were limited to LTS and age; therefore, the potential effects of sex and other unmeasured clinical, anatomical, or surgical confounders cannot be excluded. In addition, the marked predominance of male patients in the study population may limit the generalizability of the findings to female patients.

### 4.4. Measurement Reliability and Observer Effects

The Hudek method has previously been reported to demonstrate high measurement repeatability [10,11]. In the present study, intraobserver repeatability was excellent for the radiologist (ICC = 0.985; 95% CI, 0.975–0.990) and good for the orthopedic surgeon (ICC = 0.852; 95% CI, 0.769–0.906). In contrast, interobserver agreement between the radiologist and orthopedic surgeon remained moderate, with ICC values of 0.514 for the first measurements, 0.541 for the second measurements, and 0.547 when the mean of the two repeated measurements was used. These findings indicate that an observer’s ability to reproduce their own measurements with high consistency does not necessarily imply that different observers will obtain the same absolute LTS values.

Bland–Altman analysis showed that the orthopedic surgeon’s measurements were, on average, 0.85° higher than those of the radiologist, with 95% limits of agreement ranging from −6.40° to +4.70° (radiologist minus orthopedic surgeon). No significant proportional bias between the measurement difference and measurement magnitude was detected (*p* = 0.116). However, because a clinically acceptable measurement difference was not predefined, it cannot be concluded that the measurements obtained by the two observers are interchangeable. The observed differences despite the use of the same measurement method suggest that observer-related variability may arise in the identification of anatomical landmarks and placement of the plateau tangent [6,17]. Importantly, the relatively wide limits of agreement are substantial when considered in relation to the magnitude of the exploratory thresholds identified in this study. Such between-observer variability could materially affect whether an individual patient is classified above or below a proposed threshold. Therefore, absolute LTS measurements obtained by different observers should be interpreted cautiously when applying threshold-based classifications.

The relationship between this finding and the derived thresholds is also noteworthy. Although the orthopedic surgeon’s measurements were, on average, 0.85° higher than those of the radiologist, the exploratory Youden threshold derived from the orthopedic surgeon’s measurements was also approximately 1.1° higher than that derived from the radiologist’s measurements (5.4° vs. 4.3°). The concordant direction of these findings suggests that systematic observer-related differences in measurement may contribute to differences in thresholds derived from the same dataset. However, the study was not designed to directly test this relationship, confidence intervals for the thresholds themselves were not calculated, and the AUCs of the two observers were not statistically compared. Therefore, this observation should be considered hypothesis-generating. Nevertheless, it suggests that, when interpreting LTS thresholds reported across different studies, observer-related measurement characteristics may need to be considered alongside differences in study populations and imaging or measurement methods.

### 4.5. Strengths

One of the important strengths of this study is that LTS measurements were performed on MRI examinations obtained before primary ACL reconstruction, thereby avoiding the potential effects of postoperative tunnels and implants on anatomical measurements. In addition, only patients with definitively classified graft rerupture were included in the rerupture group, whereas cases considered suspicious or those that could not be definitively classified based on the combined clinical and imaging findings were excluded.

Additional methodological strengths aimed at reducing observer bias included anonymization of the MRI examinations, presentation of images from the rerupture and control groups in mixed order, and prevention of observer access to clinical records, follow-up outcomes, and group allocation. Both observers measured all cases in two separate assessment rounds, with the second assessment performed four weeks later without access to the initial measurement results. Furthermore, the inclusion of a radiologist and an orthopedic surgeon from different specialties, together with the assessment of measurement reliability using both ICC and Bland–Altman methods, enabled evaluation not only of the repeatability of LTS measurements but also of absolute agreement between observers.

The statistical power of the available sample was calculated to be 91.5% based on a Cohen’s *d* effect size of 0.863 derived from Jaecker et al. [6]. This value reflects the ability of the available sample to detect a between-group difference in the magnitude reported in the reference study and should not be interpreted as evidence of the precision of the exploratory ROC thresholds or the adequacy of all secondary analyses.

### 4.6. Limitations

This study has several limitations. First, its retrospective, single-center design introduces the potential for selection bias and residual confounding by unmeasured factors. Control eligibility required both the availability of pre-reconstruction MRI in our institutional imaging archive and at least two years of documented postoperative follow-up. These requirements may have preferentially included patients with more complete institutional imaging and longitudinal follow-up, thereby introducing selection bias and potentially limiting the representativeness of the control group and the generalizability of the findings. Although controls were not individually matched to rerupture cases, they were randomly selected from eligible patients aged 18–40 years by lot drawing to achieve the prespecified 1:2 case-to-control ratio.

Second, the limited number of patients in the rerupture group (*n* = 23) restricts the statistical precision of the ROC-derived thresholds and regression estimates, as reflected by their confidence intervals, and limits the generalizability of these findings. The retrospective power calculation addressed only the primary between-group comparison of LTS and should not be interpreted as demonstrating adequate precision for the ROC, regression, or reliability analyses. Of the 23 patients classified as having definite graft rerupture, 20 underwent revision ACL reconstruction, including four whose revision procedures were performed at another institution and documented through the Turkish national electronic health record system. The remaining three patients had recurrent symptomatic instability, clinical examination findings consistent with graft failure, and MRI findings consistent with graft rerupture; revision ACL reconstruction was recommended, but these patients declined surgery. Because surgical confirmation was unavailable in these three patients, some potential for outcome misclassification cannot be completely excluded.

Third, other potential causes of graft failure, such as major reinjury and tunnel malposition, were not systematically evaluated in all patients using predefined objective criteria. Therefore, the association between LTS and rerupture cannot be considered fully independent of these factors. Similarly, the multivariable models were limited to LTS and age, and other potential anatomical, clinical, and surgical confounders were not evaluated. The marked predominance of male patients in the study population also limits the generalizability of the findings to female patients. In addition, because only patients aged 18–40 years were included, the findings may not be generalizable to younger or older patients.

Finally, the LTS thresholds identified in the ROC analyses were derived using the Youden index within the same study cohort and were not tested in an independent validation cohort. The relatively wide confidence intervals around the sensitivity estimates, together with the absence of confidence intervals for the thresholds themselves, further limit the precision of these values. In addition, although the two observers represented different specialties, the measurement analyses were based on only two observers. Therefore, the findings regarding interobserver variability may have limited generalizability to observers with different levels of experience and to larger groups of observers.

## 5. Conclusions

Higher LTS measured using the Hudek method on MRI obtained before primary ACL reconstruction was associated with graft rerupture, and this association remained significant after adjustment for age for both observers. Although our findings support the previous literature reporting an association between increased LTS and graft failure, the lower exploratory thresholds identified in the present study and the systematic measurement difference between observers suggest that a single LTS threshold may not be directly applicable across different populations and observers. Given the high specificity but limited sensitivity of the exploratory thresholds and their derivation from the same cohort, these values should not be regarded as validated clinical decision thresholds. The role of LTS in graft rerupture risk assessment and the observer-related variability in derived thresholds require validation in independent, larger, and preferably prospective multicenter cohorts.

## Figures and Tables

**Table 1:** Comparison of demographic and clinical characteristics between the rerupture and control groups.

Characteristics	Rerupture Group (*n* = 23)	Control Group (*n* = 46)	*p* Value
Age, median (Q1–Q3), years	28.00 (23.00–40.00)	37.00 (29.00–40.00)	0.103
Sex, *n* (%)			
Male	22 (95.7)	38 (82.6)	0.254
Female	1 (4.3)	8 (17.4)	
Side, *n* (%)			
Right	15 (65.2)	24 (52.2)	0.303
Left	8 (34.8)	22 (47.8)	
Concomitant surgical procedure, *n* (%)			
Isolated ACL reconstruction	17 (73.9)	31 (67.4)	0.949
Meniscectomy	1 (4.3)	2 (4.3)	
Meniscal repair	4 (17.4)	11 (23.9)	
Microfracture	1 (4.3)	2 (4.3)	

**Table 2:** Comparison of LTS measurements between the rerupture and control groups.

Measurements	Rerupture Group (*n* = 23) Mean ± SD	Control Group (*n* = 46) Mean ± SD	*p* Value
Radiologist, first measurement	4.89 ± 3.61	1.34 ± 2.42	<0.001
Radiologist, second measurement	4.75 ± 3.34	1.37 ± 2.57	<0.001
Orthopedic surgeon, first measurement	5.03 ± 3.46	2.81 ± 2.33	0.009
Orthopedic surgeon, second measurement	4.61 ± 3.16	2.45 ± 2.41	0.002

**Table 3:** Intraobserver repeatability of LTS measurements.

Observer	ICC	95% CI	*p* Value
Radiologist	0.985	0.975–0.990	<0.001
Orthopedic surgeon	0.852	0.769–0.906	<0.001

**Table 4:** Interobserver agreement between the radiologist and orthopedic surgeon.

Comparison	ICC	95% CI	*p* Value
First measurements	0.514	0.311–0.671	<0.001
Second measurements	0.541	0.352–0.688	<0.001
Mean of the two repeated measurements	0.547	0.355–0.694	<0.001

**Table 5:** ROC analysis of the ability of mean LTS measurements to discriminate between the rerupture and control groups.

Observer	AUC (95% CI)	*p*-Value	Cutoff (°)	Sensitivity, % (95% CI)	Specificity, % (95% CI)	PPV (%)	NPV (%)	Youden Index
Radiologist	0.794 (0.678–0.910)	<0.001	≥4.3	60.9 (38.5–80.3)	93.5 (82.1–98.6)	82.4	82.7	0.543
Orthopedic surgeon	0.700 (0.563–0.837)	0.007	≥5.4	43.5 (23.2–65.5)	91.3 (79.2–97.6)	71.4	76.4	0.348

AUC: area under the receiver operating characteristic curve; CI: confidence interval; PPV: positive predictive value; NPV: negative predictive value. Cutoff values were determined based on the highest Youden index in the study dataset. PPV and NPV depend on the prevalence of rerupture in the study sample and therefore may not be directly generalizable to populations with different rerupture rates.

**Table 6:** Logistic regression analysis of factors associated with graft rerupture.

Model	Variable	Adjusted OR	95% CI	*p* Value
Model 1: Radiologist	Mean of two LTS measurements (per 1° increase)	1.682	1.241–2.280	0.001
	Age (per 1-year increase)	0.930	0.860–1.006	0.071
Model 2: Orthopedic surgeon	Mean of two LTS measurements (per 1° increase)	1.412	1.123–1.776	0.003
	Age (per 1-year increase)	0.923	0.856–0.995	0.036

OR: odds ratio; CI: confidence interval.

## Data Availability

The data supporting the findings of this study are available from the corresponding author upon reasonable request, subject to applicable ethical and institutional restrictions.

## Abbreviations

ACL, anterior cruciate ligament; ACLR, anterior cruciate ligament reconstruction; AUC, area under the receiver operating characteristic curve; CI, confidence interval; ICC, intraclass correlation coefficient; LTS, lateral tibial slope; MRI, magnetic resonance imaging; NPV, negative predictive value; OR, odds ratio; PPV, positive predictive value; ROC, receiver operating characteristic.
